# Predicting acute and late toxicity in prostate cancer stereotactic ablative radiotherapy: the role of dosimetric parameters and prostate volume

**DOI:** 10.1007/s00066-024-02343-2

**Published:** 2025-01-10

**Authors:** Gokhan Ozyigit, Pervin Hurmuz, Pantea Bayatfard, Burak Tilki, Yagiz Yedekci, Melek Tugce Yilmaz

**Affiliations:** Department of Radiation Oncology, Hacettepe University Faculty of Medicine, Ankara, Turkey

**Keywords:** Prostate cancer, SABR, Toxicity, Prostate volume, Dosimetry

## Abstract

**Purpose:**

Our objective was to identify the dosimetric parameters and prostate volume that most accurately predict the incidence of acute and late gastrointestinal (GI) and genitourinary (GU) toxicity in prostate cancer stereotactic ablative radiotherapy (SABR) treatments.

**Methods:**

We conducted a retrospective analysis of 122 patients who received SABR for prostate cancer at our clinic between March 2018 and September 2022 using a five-fraction SABR regimen. The existing plans of these patients were re-evaluated according to our institutional protocols (Hacettepe University [HU-1] and HU-2) as well as PACE‑B, RTOG 0938, and NRG GU005 dose–volume constraints. Univariate and multivariate logistic regression analyses were performed using SPSS version 23.0 (IBM, Armonk, NY, USA).

**Results:**

The median follow-up was 24.7 months (0.8–94.4 months). For acute GU toxicity, moderate-dose regions were predictive for grade 1–2 toxicity, while high-dose regions were more associated with grade 3–4 toxicity. For late GU toxicity, moderate–high-dose regions were predictive. For GI toxicity, moderate-dose regions were important for both acute and late toxicity. The HU protocol encompassed all significant dosimetric factors influencing toxicity outcomes. A prostate volume threshold of 60 cc was predictive of acute grade 3–4 GU toxicity.

**Conclusion:**

Our study highlighted the critical role of moderate-dose regions for acute and late GI and GU toxicity. Prostate treatment plans should be rigorously evaluated, and moderate doses should be minimized. The HU protocol is an eligible choice for five-fraction SABR plans.

**Supplementary Information:**

The online version of this article (10.1007/s00066-024-02343-2) contains supplementary material, which is available to authorized users.

## Introduction

Stereotactic ablative radiotherapy (SABR) is a well-established treatment method in prostate cancer [1–4]. Its lower α/β value (< 1.5) compared to the surrounding organs at risk (OARs) leads to an increase in the therapeutic ratio when large fraction doses are applied [5]. The steep dose gradient provides maximum sparing for the rectum and bladder, and short schedules ensure rapid treatment completion. Hence, SABR is a viable option for treating prostate cancer. Although OARs are spared to the maximum due to the sophisticated nature of the technique, more toxicity is encountered with SABR, especially acute toxicity, as compared to other conventional and moderately hypofractionated regimens [6, 7]. To avoid this, a thorough evaluation of the treatment plan is crucial.

The recent introduction of SABR into everyday clinical practice has resulted in a paucity of data on the most effective dosimetric factors for predicting toxicity. Several studies have used various OAR dose limits in the literature, and it remains unclear which dose limits more accurately reflect toxicity [8–10]. Our center has been using SABR for prostate cancer treatment for 17 years. We have implemented OAR dose–volume constraints specific to our clinic (see Supplementary Table 1) and we have previously reported oncological and toxicity outcomes [11, 12].^x, x^.

The correlation between prostate volume and toxicity is mostly based on data obtained from brachytherapy experience [13, 14]. Specifically, the commonly used volume limit of 50 cc is an ambiguous threshold that is applicable to brachytherapy treatments and may lead to the unnecessary use of neoadjuvant androgen deprivation therapy (ADT) in external radiotherapy (RT) methods. There are studies indicating that the prostate volume may be related to toxicity in three-dimensional (3D) conformal RT and intensity-modulated RT (IMRT) [15, 16]. However, there are no established data indicating that the prostate volume increases the toxicity of SABR. In fact, there are even reports that satisfactory outcomes can be obtained even in individuals with larger prostate volumes [17].

For this study, we evaluated our own clinical protocols (Hacettepe University‑1 [HU-1] and HU-2) along with the dose–volume parameters of commonly used protocols (PACE‑B, RTOG 0938, and NRG GU005) to identify the dosimetric parameters that best predict the occurrence of acute and late gastrointestinal (GI) and genitourinary (GU) toxicity in prostate SABR. We also investigated the effect of prostate volume on toxicity, as well as the optimal threshold.

## Materials and methods

### Patient cohort

We retrospectively evaluated 122 patients who received SABR for biopsy-confirmed prostate cancer at our clinic between March 2018 and September 2022. Since 2007, we have implemented several image-guided RT (IGRT) approaches in our treatment with SABR, such as fiducial tracking using kilovoltage (kV) or megavoltage cone-beam computed tomography (MV-CBCT) images. In this study, we only included patients treated with the same treatment technique using MV-CBCT IGRT without fiducials. We included patients aged ≥ 18 years diagnosed with an T1-3N0M0 tumor according to the American Joint Commission on Cancer (AJCC) 8th edition and with a Karnofsky performance score (KPS) ≥ 70; we excluded patients with distant metastasis, lymph node involvement, previous pelvic RT, previous transurethral resection, and a prior diagnosis of cancer. The risk category was determined using the D’Amico classification [18]. The study was approved by the Hacettepe University Ethics Committee of Non-Invasive Clinical Research with registration number GO 23/316 on April 18, 2023.

### Treatment

**Fig. 1:**
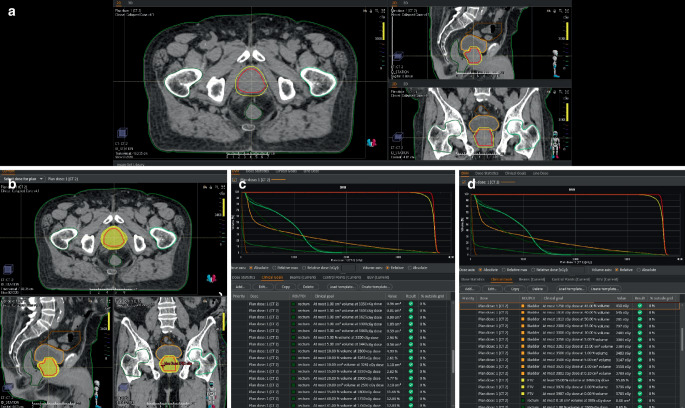
Details of a typical prostate cancer stereotactic ablative radiotherapy plan at our institution for a 60-year-old patient with intermediate-risk prostate cancer treated with 5 fractions of 730 cGy every other day. **a** Contours: the clinical target volume (CTV) encompasses the entire prostate; the planning target volume (PTV) is created by expanding the CTV by 4 mm in the craniocaudal, anterior, and lateral directions, and by 3 mm posteriorly. Red delineation: CTV; yellow delineation: PTV; green delineation: rectum. **b** Treatment plan: the yellow dose color wash represents the 95% isodose line. **c** and **d** Dose–volume histogram and dose–volume constraints according to the HU protocol

Patients were advised on bladder and rectum preparation before simulation computed tomography (sCT). To ensure consistent bladder filling, all patients were instructed to drink 500 ml of water and wait for 30 min prior to undergoing sCT. The target rectal width in sCT was set to be less than 40 mm as an institutional protocol. All patients underwent sCT without intravenous contrast with a 1.25 mm slice thickness using the Toshiba Aquilion LB CT Simulator (Toshiba Medical Systems, Otawara, Japan). The RayStation (RaySearch Laboratories, Stockholm, Sweden) version 8A treatment planning system was used for contouring and treatment planning (Fig. 1).

Patients classified as low and intermediate risk had a clinical target volume (CTV) that included the whole prostate, whereas high-risk patients had a CTV that included both the entire prostate and the proximal 1 cm of the seminal vesicles. If the seminal vesicle was invaded by the tumor, whole vesicles were included in the treatment field. We performed magnetic resonance image (MRI) fusion on patients with MRI. The planning target volume (PTV) was created by expanding the CTV by 4–5 mm craniocaudally, anteriorly, and laterally, and by 3 mm posteriorly. Prophylactic lymphatic irradiation was not administered as per clinical protocol. All patients were prescribed 36.5 Gy in 5 fractions every other day using an Elekta Versa HD® (Elekta AB, Stockholm, Sweden). Rectal and bladder fullness were assessed using the MV-CBCT in each fraction. We aimed for 95% of the CTV to receive 100% of the prescribed dose and for 95% of the PTV to receive at least 95% of the prescribed dose. Figure 1 displays a typical contouring, plan, and dose–volume histogram (DVH). The OAR dose volume constraints employed for the SABR plan in our clinic are given in Supplementary Table 1.

**Table 1:** Baseline characteristics of the patient cohort

Characteristics	*n* (%) (*N* = 122)
*Age (median)*	72 years (range 20–91)
*Gleason score*	
3 + 3	36 (29.5)
3 + 4	29 (23.5)
4 + 3	23 (19)
3 + 5	3 (2.5)
4 + 4	13 (10.7)
4 + 5	11 (9)
5 + 4	2 (1.7)
5 + 5	5 (4.1)
*T classification*^*a*^	
T1	4 (3.3)
T2a	37 (30.3)
T2b	20 (15.6)
T2c	33 (27.2)
T3a	28 (23.6)
*D’Amico risk classification*	
Low	22 (18)
Intermediate	40 (32.8)
High	60 (49.2)
*Adjuvant ADT*	
Yes	99 (80.8)
No	23 (19.2)
*Neoadjuvant ADT*	
Yes	48 (39.3)
No	74 (60.7)

^a^According to American Joint Committee on Cancer (AJCC) 8th edition

*ADT* androgen deprivation therapy

We recommended ADT for 6 months in patients with unfavorable intermediate-risk disease and for 24 months in patients with high-risk disease. There was no specific prostate volume limit that would hamper treatment; however, patients who did not meet the institutional OAR constraints as per HU‑1 and HU‑2 received neoadjuvant ADT for 3–6 months. We started ADT and SABR concurrently, except for in these patients.

### Endpoints and follow-up

Patients were evaluated during treatment and at 3‑month intervals following treatment. We graded and recorded acute and late GI and GU toxicities using the Radiation Therapy Oncology Group (RTOG) toxicity criteria [19]. All patients were engaged in a rigorous monitoring program, and the senior resident and professors in charge of the prostate cancer outpatient clinic (G.O. and P.H.) conducted assessments of all adverse events and their severity. Prostate-specific antigen (PSA) was assessed every 3 months during the initial 2 years following treatment, every 6 months for 2–5 years, and annually after 5 years. The toxicity events observed within 90 days of SABR were classified as acute toxicity, whereas those observed thereafter were classified as late toxicity. Toxicity and dosimetric correlations were evaluated in two distinct categories for each side effect type (GI and GU): acute and chronic. And again, each group was further stratified into two categories based on the severity of toxicity as grade 1–2 (mild–moderate) and grade 3–4 (severe).

The existing plans of 122 patients treated in our clinic were re-evaluated according to our institutional protocol (HU‑1 and HU-2) and PACE‑B, RTOG 0938, and NRG GU005 (See Supplementary Table 1) [8–10]. We examined the correlation between dosimetric parameters in these protocols and acute and late GI and GU toxicities.

### Statistical analysis

Statistical analyses were performed using the Statistical Package for the Social Sciences (SPSS) version 23.0 (IBM, Armonk, NY, USA). Descriptive analyses were presented using medians and minimum–maximum values. The Mann–Whitney U test was used to investigate the effect of prostate volume on toxicity. Univariate logistic regression analysis (UVA) was conducted to search for significant dosimetric predictors of acute and late GI/GU toxicity, and the *p*-value, odds ratio (OR), and the 95% confidence intervals (CI) of the OR were reported. The possible factors identified in univariate analyses (*p* < 0.1) were further included in the multivariate logistic regression analyses (MVA). Dosimetric variables that are significant for toxicity in MVA were further analyzed using receiver operating characteristic (ROC) analysis. When significant cut-off values were found with Youden’s index, the sensitivity, specificity, and positive and negative predictive values were presented. A *p*-value < 0.05 was considered statistically significant.

## Results

The median age was 72 years (range 20–91). The median prostate-specific antigen (PSA) level at diagnosis was 10 ng/mL (range 2.3–324 ng/mL), and the median Gleason score (GS) was 7 (range 6–10). Table 1 presents the detailed baseline characteristics of the patients.

Neoadjuvant ADT was used in 48 patients (39.3%), with a median duration of 4 months (range 1–60 months). Eight patients received neoadjuvant ADT for more than 6 months, and these patients were referred to our clinic after ADT had been initiated. A total of 98 patients (80.3%) used concurrent adjuvant ADT, with a median duration of 9 months (3–30 months).

In follow-ups, the median PSA nadir was 0.01 ng/mL (range 0.001–0.795 ng/mL). The median follow-up was 24.7 months (0.8–94.4 months), and the median PSA at the last follow-up was 0.09 ng/mL (0–3.23 ng/mL). At last follow-up, 109 patients were alive without disease (89.3%), and 8 patients had recurrence (6.6%). Five patients (4%) died due to reasons other than the disease. Two of the 8 patients who developed recurrence experienced distant metastasis, while others had locoregional recurrence. Patients had a 2- and 5‑year progression-free survival (PFS) of 95% and 82%, respectively.

### Toxicity outcomes

Acute GU toxicity was observed in 81 patients (66.4%). Sixty-five (53.2%) patients experienced grade 1–2 toxicity, and 16 (13.1%) patients experienced grade 3–4 toxicity. The most common GU side effects were dysuria in 67 patients (54.9%), nocturia in 32 (26.2%) patients, and hematuria in 4 patients (3.2%). Acute GI toxicity was observed in 52 patients (42.6%). Grade 1–2 toxicity was observed in 39 (31.9%) patients, and grade 3–4 toxicity was observed in 13 (10.6%). The most common GI side effects were proctitis in 43 patients (35.2%), hematochezia in 7 patients (5.7%), and diarrhea in 4 patients (3.2%).

Late GU toxicity was observed in 33 patients (27%). Twenty-nine patients (23.7%) had grade 1–2 toxicity, and 4 patients (3.2%) experienced grade 3–4 toxicity. The most common GU late side effects were dysuria in 18 patients (14.7%), incontinence in 10 patients (8.1%), nocturia in 8 patients (6.5%), hematuria in 5 patients (4%), and urinary stricture in 2 patients (1.6%). Of the 10 patients who developed incontinence, 4 experienced persistent incontinence at the 24-month mark, whereas the other 6 patients’ incontinence was resolved (see Supplementary Fig. 1). Late GI toxicity was observed in 16 patients (13.1%). Fourteen patients (11.4%) experienced grade 1–2 toxicity, while 2 patients (1.6%) experienced grade 3–4 toxicity. The most common late GI side effects were hematochezia in 9 (7%) patients, chronic proctitis in 6 patients (4.9%), and chronic diarrhea in 1 patient (0.8%). Of the patients who had hematochezia, 2 had grade 3–4 hematochezia. We referred these patients to argon laser treatment, and at 12 months, one patient’s hematochezia had resolved (see Supplementary Fig. 1).

### Dosimetric correlations with toxicity

The median (range; min–max) values of the dose–volume constraints for the rectum and bladder are presented in Supplementary Table 2.

For acute GU toxicity, dose received by 15 cc of the bladder volume (D15cc) was a significant predictor for grade 3–4 toxicity in MVA (*p* = 0.04). Furthermore, dose received by 10% and 1% of the bladder volume (D10% and D1%) showed a trend towards significance and were evaluated as clinically relevant predictors for grade 1–2 and grade 3–4 GU toxicity, respectively (*p* = 0.07 and *p* = 0.05; Table 2). We further entered these predictors into ROC analysis to determine the optimal threshold (see Supplementary Fig. 2). The optimum cut-off for D15cc was 26.3 Gy, with 62% sensitivity and 58% specificity (*p* = 0.04, area under curve [AUC]: 0.657, 95% confidence interval [CI]: 0.541–0.774). The optimum cut-off for D10% was 19.08 Gy, with 61% sensitivity and specificity (*p* = 0.02, AUC: 0.623, 95% CI: 0.523–0.0723) and the optimal cut-off for D1% was 35.4 Gy, with 56% sensitivity and 56% specificity (*p* = 0.07, AUC: 0.640, 95% CI: 0.518–0.763).

**Table 2:** Uni- and multivariate logistic regression analysis of acute genitourinary toxicity and dose–volume histogram parameters for bladder

	Acute grade 1–2 GU toxicity				Acute grade 3–4 GU toxicity			
	UVA		MVA^a^		UVA		MVA^a^	
*DVH parameters for bladder*	*OR (95% CI)*	*p-*value	*OR (95% CI)*	*p-*value	*OR (95% CI)*	*p-*value	*OR (95%CI)*	*p-*value
*D1%*	1.582 (1.073–2.332)	*0.02*	1.511 (0.909–2512)	0.1	0.548 (0.300–1.004)	*0.05*	0.857 (0.736–0.999)	*0.05*
*D5%*	1.087 (1.010–1.170)	*0.02*	0.805 (0.576–1.127)	0.2	0.929 (0.826–1.044)	0.2	–	
*D10%*	1.075 (1.009–1.145)	*0.02*	1.494 (0.959–2.327)	*0.07*	0.938 (0.851–1.033)	0.1	–	
*D35%*	1.081 (0.979–1.193)	0.1	–		0.137 (0.791–1.033)	0.1	–	
*D50%*	1.074 (0.920–1.253)	0.3	–		0.945 (0.764–1.167)	0.5	–	
*D0.1cc*	0.898 (0.511–1.579)	0.7	–		0.746 (0.324–1.718)	0.4	–	
*D1cc*	0.933 (0.562–1.549)	0.7	–		0.673 (0.303–1.493)	0.3	–	
*D15cc*	1.006 (0.929–1.089)	0.8	–		0.863 (0.747–0.997)	*0.04*	0.857 (0.736–0.999)	*0.04*
*V17.5Gy*	1.058 (0.990–1.132)	*0.09*	0.371 (0.033–4.156)	0.4	0.949 (0.865–1.040)	0.2	–	
*V18.1Gy*	1.063 (0.990–1.140)	*0.09*	6.446 (0.071–585.213)	0.4	0.946 (0.859–1.042)	0.2	–	
*V18.12Gy*	1.062 (0.990–1.140)	*0.09*	0.353 (0.008–15.139)	0.5	0.946 (0.859–1.042)	0.2	–	
*V35Gy*	1.084 (0.888–1.324)	0.4	–		0.569 (0.691–1.226)	0.5	–	
*V37Gy*	0.765 (0.382–1.532)	0.4	–		0.891 (0.363–2.186)	0.8	–	

The *p*-values in italics represent those of univariate analysis (UVA) for the variables included in the multivariate analysis (MVA). For MVA, the italicized *p*-values correspond to variables that were considered statistically and clinically significant.

*GU* genitourinary, *UVA* univariate analysis, *MVA* multivariate analysis, *OR* odds ratio, *CI* confidence interval, *DVH* dose–volume histogram, *Dx%* dose received by x% of the volume, *Dxcc* dose received by x cc of the volume, *VxGy* volume receiving a dose X ≥ Gy

^a^Dosimetric parameteres that had *p* < 0.1 in UVA included in MVA

For acute GI toxicity, none of the dosimetric parameters were predictive for grad 1–2 toxicity in UVA and MVA (Table 3). For acute grade 3–4 GI toxicity, on the other hand, rectum volume receiving a dose of ≥18 Gy  (V18Gy) was a significant predictor, and rectum V18.12Gy was also clinically relevant (*p* = 0.04 and *p* = 0.05, respectively). The optimum threshold for V18Gy was 23.02%, with 61% sensitivity and 57% specificity (*p* = 0.1, AUC: 0.636, 95% CI: 0.449–0.822) and for V18.12Gy, the optimum threshold was 22.8%, with 61% sensitivity and 57% specificity (*p* = 0.1, AUC: 0.634, 95% CI: 0.447–0.821).

**Table 3:** Univariate and multivariate logistic regression analysis of acute gastrointestinal toxicity and dose–volume histogram parameters for rectum

	Acute grade 1–2 GI toxicity				Acute grade 3–4 GI toxicity			
	UVA		MVA^a^		UVA		MVA^a^	
DVH parameters for rectum	OR (95% CI)	*p-*value	OR (95% CI)	*p-*value	OR (95% CI)	*p-*value	OR (95% CI)	*p-*value
*D1%*	0.757 (0.502–1.140)	0.1	–		0.757 (0.502–1.140)	0.2	–	
*D10%*	0.939 (0.827–1.066)	0.3	–		0.899 (0.732–1.103)	0.3	–	
*D15%*	0.960 (0.858–1.073)	0.4	–		0.859 (0.709–1.041)	0.1	–	
*D20%*	0.968 (0.870–1.078)	0.5	–		0.864 (0.719–1.039)	0.1	–	
*D40%*	0.980 (0.885–1.086)	0.7	–		0.850 (0.720–1.004)	*0.05*	2.247 (0.839–6.021)	0.1
*D50%*	0.993 (0.900–1.095)	0.8	–		0.857 (0.733–1.002)	*0.05*	0.608 (0317–1.165)	0.1
*D0.1cc*	0.902 (0.518–1.571)	0.7	–		1.215 (0.539–2.737)	0.6	–	
*D1cc*	0.994 (0.728–1.358)	0.9	–		1.081 (0.700–1.669)	0.7	–	
*D3cc*	1.052 (0.891–1.243)	0.5	–		0.914 (0.688–1.214)	0.5	–	
*D5cc*	1.044 (0.924–1.179)	0.4	–		0.940 (0.766–1.154)	0.5	–	
*D10cc*	1.048 (0.952–1.155)	0.3	–		0.886 (0.745–1.053)	0.1	–	
*D20cc*	1.067 (0.970–1.173)	0.1	–		0.846 (0.715–1.002)	*0.05*	0.825 (0.645–1.056)	0.1
*V17.5Gy*	0.982 (0.939–1.027)	0.4	–		0.926 (0.866–0.991)	*0.02*	2.392 (0.596–9.603)	0.2
*V18Gy*	0.979 (0.932–1.028)	0.4	–		0.919 (0.853–0.989)	*0.02*	0.003 (0–0.970)	*0.04*
*V18.12Gy*	0.979 (0.932–1.030)	0.4	–		0.918 (0.852–0.990)	*0.02*	118.894 (0.911–15518)	*0.05*
*V28Gy*	0.872 (0.759–1.002)	*0.05*	0.900 (0.748–1.081)	0.2	0.944 (0.800–1.113)	0.4	–	
*V29Gy*	0.892 (0.749–1.061)	0.1	–		0.877 (0.683–1.127)	0.3	–	
*V32Gy*	0.825 (0.642–1.058)	0.1	–		0.974 (0.670–1.417)	0.8	–	
*V32.63Gy*	0.793 (0.609–1.033)	*0.08*	0.919 (0.634–1.332)	0.6	1.045 (0.693–1.577)	0.8	–	
*V33Gy*	1.004 (0.605–1.666)	0.9	–		1.307 (0.583–2.931)	0.5	–	
*V33.5Gy*	0.973 (0.543–1.744)	0.9	–		0.574 (0.518–3.283)	0.5	–	
*V34.4Gy*	0.868 (0.374–2.015)	0.7	–		1.978 (0.458–8.537)	0.3	–	
*V36Gy*	0.677 (0.048–9.571)	0.7	–		2.253 (0.023–221.715)	0.7	–	

The *p*-values in italics represent those of univariate analysis (UVA) for the variables included in the multivariate analysis (MVA). For MVA, the italicized *p*-values correspond to variables that were considered statistically and clinically significant.

*GI* gastrointestinal, *UVA* univariate analysis, *MVA* multivariate analysis, *OR* odds ratio, *CI* confidence interval, *DVH* dose–volume histogram, *Dx%* dose received by x% of the volume, *Dxcc* dose received by x cc of the volume, *VxGy* volume receiving a dose X ≥ Gy

^a^Dosimetric parameters with *p* < 0.1 in UVA were included in MVA

For late GU toxicity, the only predictor for grade 1–2 toxicity that was marginally significant was bladder D1% (*p* = 0.06) and for grade 3–4 toxicity, bladder D15cc (*p* = 0.09) in UVA. Therefore, we were unable to conduct MVA (Table 4). The optimal cut-off for D1% was 35.3 Gy, with 51% sensitivity and 51% specificity (*p* = 0.6, AUC: 0.525, 95% CI: 0.393–0.657) and for D15 cc, the optimal cut-off was 28.9 Gy, with 75% sensitivity and 77% specificity (*p* = 0.1, AUC: 0.738, 95% CI: 0.464–1.000).

**Table 4:** Univariate and multivariate logistic regression analysis of late genitourinary toxicity and dose–volume histogram parameters for bladder

	Late grade 1–2 GU toxicity				Late grade 3–4 GU toxicity			
	UVA		MVA^a^		UVA		MVA^a^	
DVH parameters for bladder	OR (95% CI)	*p-*value	OR (95% CI)	*p-*value	OR (95% CI)	*p-*value	OR (95% CI)	*p-*value
*D1%*	1.420 (0.977–2.062)	*0.06*	–		0.697 (0.376–1.293)	0.2	–	
*D5%*	1.045 (0.974–1.122)	0.2	–		0.946 (0.836–1.070)	0.3	–	
*D10%*	1.009 (0.947–1.076)	0.7	–		0.949 (0.856–1.053)	0.3	–	
*D35%*	0.946 (−854–1.047)	0.2	–		0.897 (0.777–1.036)	0.8	–	
*D50%*	0.907 (0.774–1.063)	0.2	–		0.972 (0.765–1.234)	0.8	–	
*D0.1cc*	0.961 (0.526–1.756)	0.8	–		0.785 (0.316–1.952)	0.6	–	
*D1cc*	1.266 (0.737–2.173)	0.3	–		0.705 (0.297–1.674)	0.4	–	
*D15cc*	1.063 (0.976–1.157)	0.1	–		0.876 (0.750–1.022)	*0.09*	–	
*V17.5Gy*	0.988 (0.922–1.059)	0.7	–		0.946 (0.856–1.045)	0.2	–	
*V18.1Gy*	0.988 (0.919–1.062)	0.7	–		0.944 (0.850–1.048)	0.2	–	
*V18.12Gy*	0.987 (0.918–1.062)	0.7	–		0.943 (0.849–1.047)	0.2	–	
*V35Gy*	1.114 (0.932–1.454)	0.1	–		0.901 (0.660–1.231)	0.5	–	
*V37Gy*	0.855 (0.435–1.677)	0.6	–		1.047 (0.350–3.133)	0.1	–	
*V38.06Gy*	0.002 (0–21379)	0.4	–		18.064 (0–76)	0.7	–	

The *p*-values in italics represent those of univariate analysis (UVA) for the variables included in the multivariate analysis (MVA). For MVA, the italicized *p*-values correspond to variables that were considered statistically and clinically significant.

*GU* genitourinary, *UVA* univariate analysis, *MVA* multivariate analysis, *OR* odds ratio, *CI* confidence interval, *DVH* dose–volume histogram, *Dx%* dose received by x% of the volume, *Dxcc* dose received by x cc of the volume, *VxGy* volume receiving a dose X ≥ Gy

^a^Dosimetric parameters with *p* < 0.1 in UVA were included in MVA

For late-grade 1–2 GI toxicity, rectum V28Gy was the only predictor that approached statistical significance (*p* = 0.06). None of the rectum dosimetric parameters were significant for late grade 3–4 GI toxicity (Table 5). This could be attributed to a lack of occurrences. The optimal cut-off for V28Gy was found to be 9.6%, with 57% sensitivity and 85% specificity (*p* = 0.2, AUC: 0.592, 95% CI: 0.376–0.808).

**Table 5:** Univariate and multivariate logistic regression analysis of late gastrointestinal toxicity and dose–volume histogram parameters for rectum

	Late grade 1–2 GI toxicity				Late grade 3–4 GI toxicity			
	UVA		MVA^a^		UVA		MVA^a^	
DVH parameters for rectum	OR (95% CI)	*p-*value	OR (95% CI)	*p-*value	OR (95% CI)	*p-*value	OR (95% CI)	*p-*value
*D1%*	0.751 (0.409–1.378)	0.3	–		0.506 (0.106–2.416)	0.3	–	
*D10%*	1.023 (0.857–1.221)	0.8	–		1.092 (0.716–1.666)	0.6	–	
*D15%*	0.946 (0.800–1.118)	0.5	–		1.088 (0.743–1.593)	0.6	–	
*D20%*	0.970 (0.828–1.137)	0.7	–		1.070 (0.734–1.558)	0.7	–	
*D40%*	0.983 (0.847–1.142)	0.8	–		1.027 (0.704–1.499)	0.8	–	
*D50%*	0.970 (0.840–1.119)	0.6	–		0.881 (0.679–1.394)	0.8	–	
*D0.1cc*	0.959 (0.427–2.154)	0.9	–		0.774 (0.096–6.241)	0.8	–	
*D1cc*	1.222 (0.832–1.795)	0.3	–		0.699 (1.152–3.214)	0.6	–	
*D3cc*	1.163 (0.934–1.448)	0.1	–		0.941 (0.480–1.846)	0.8	–	
*D5cc*	1.138 (0.968–1.338)	0.1	–		1.068 (0.708–1.611)	0.7	–	
*D10cc*	1.126 (0.985–1.287)	*0.08*	1.1181 (0.868–1.606)	0.2	1.095 (0.793–1.511)	0.5	–	
*D20cc*	1.146 (0.999–1.314)	*0.05*	1.013 (0.746–1.376)	0.9	1.082 (0.777–1.508)	0.6	–	
*V17.5Gy*	1.007 (0. 941–1.076)	0.8	–		1.070 (0.878–1.305)	0.5	–	
*V18Gy*	1.002 (0.932–1.077)	0.9	–		1.077 (0.870–1.334)	0.4	–	
*V18.12Gy*	1.000 (0.929–1.077)	0.9	–		1.077 (0.868–1.338)	0.5	–	
*V28Gy*	0.818 (0.685–0.976)	*0.02*	0.811 (0.648–1.0154)	*0.06*	1.085 (0.621–1.895)	0.7	–	
*V29Gy*	0.826 (0.649–1.052)	0.1	–		1.060 (0.546–2.056)	0.8	–	
*V32Gy*	0.770 (0.547–1.082)	0.1	–		1.010 (0.401–2.544)	0.9	–	
*V32.63Gy*	0.705 (0.499–0.995)	*0.04*	1.013 (0.545–1.882)	0.9	1.022 (0.381–2.745)	0.9	–	
*V33Gy*	0.938 (0.450–1.954)	0.8	–		0.914 (0.147–5.682)	0.9	–	
*V33.5Gy*	0.779 (0.339–1.789)	0.5	–		0.714 (0.093–5.504)	0.7	–	
*V34.4Gy*	0.615 (0.193–1.962)	0.4	–		0.447 (0.031–6.556)	0.5	–	
*V36Gy*	0.03 (0.001–0.598)	*0.02*	0.079 (0.001–10.252)	0.3	0.171 (0–312.1)	0.6	–	

The *p*-values in italics represent those of univariate analysis (UVA) for the variables included in the multivariate analysis (MVA). For MVA, the italicized *p*-values correspond to variables that were considered statistically and clinically significant.

*GI* gastrointestinal, *UVA* univariate analysis, *MVA* multivariate analysis, *OR* odds ratio, *CI* confidence interval, *DVH* dose–volume histogram, *Dx%* dose received by x% of the volume, *Dxcc* dose received by x cc of the volume, *VxGy* volume receiving a dose X ≥ Gy

^a^Dosimetric parameters with *p* < 0.1 in UVA were included in MVA

### Prostate volume and toxicity

The median prostate volume was 51.89 cc (range 19.21–124.76), and the median PTV volume was 89.97 cc (range 44.61–185.69). No relationship was found between prostate volume and overall acute and late GU toxicity (*p* = 0.1 and *p* = 0.8, respectively). When evaluated separately for acute grade 1–2 and 3–4 toxicity, the volume was shown to be statistically significant only for the latter group (*p* = 0.6 and *p* = 0.003, respectively). The prostate volume threshold that most accurately predicted acute grade 3–4 GU toxicity was 61.7 cc (*p* = 0.003, AUC: 0.729, 95% CI: 0.609–0.850), with a sensitivity of 68% and a specificity of 67%. There was no correlation between late grade 1–2 and grade 3–4 GU toxicities (*p* = 0.8 and *p* = 0.3, respectively).

There was also no correlation between prostate volume and overall acute and late GI toxicity (*p* = 0.7 and *p* = 0.7, respectively). Similarly, prostate volume did not correlate with acute grade 1–2 or grade 3–4 toxicity (*p* = 0.4 and *p* = 0.2, respectively) or with late grade 1–2 or grade 3–4 toxicity (*p* = 0.8 and *p* = 0.2, respectively).

## Discussion

In our study, we provide a comprehensive analysis of the dosimetric correlates associated with acute and late GI and GU toxicity in prostate SABR using the most commonly employed five-fraction dose–volume constraints. Our study presents an analysis of the largest single-center homogeneous series in the literature for the five-fraction treatment schedule. Studies in the literature examining the relationship between SABR dose–volume parameters and toxicity are quite heterogeneous, and many DVH recommendations currently contain conflicting results [20]. Studies have indicated that both low-to-moderate doses [21, 22] and high-dose regions [23–26] might have significance for the bladder. While rectal toxicity is often associated with high-dose regions and maximum dose parameters [23, 24, 26–28], there are studies showing the importance of moderate doses, similar to those in the bladder [24]. Again, studies utilizing a four-fraction regimen, which is not frequently used in daily practice [21, 24, 27, 29], and those focusing on a particular side effect [30, 31] do not offer sufficient guidance for our routine clinical approach. These reasons suggest that there is an ongoing requirement for well-established recommendations for prostate SABR. On the path we embarked upon for this purpose, we have not only correlated the toxicity and dose–volume constraints of commonly used protocols, but we have also validated the predictive capability of our institutional protocol in our rigorously monitored group of 122 patients.

Our toxicity outcomes were comparable to published literature [3, 4]. Regarding acute GU toxicity, our findings suggest that moderate-dose regions were important for acute 1–2 toxicity, while the high-dose regions were more associated with grade 3–4 toxicity. For late GU toxicity, moderate–high-dose regions were critical. Accordingly, the doses of bladder D10%< 19.08 Gy, D1%< 35.3 Gy, and D15cc < 26.3 Gy should be taken into consideration. Both the HU protocol and the RTOG 0938 protocol list the D10% value, recommending doses of 30 Gy and 32.63 Gy, respectively. The latter two parameters were solely present in the HU protocol, and 35 Gy for D1% and 32.62 Gy for D15 cc were recommended thresholds. These results highlight the ability of our institutional protocol to reliably forecast both acute and late GU toxicity. However, it was evident that we needed to enforce stricter criteria, particularly for the D10% parameter.

In line with our study, Alayed et al. [23] also highlighted the significance of the high-dose regions in the bladder, suggesting bladder D2cc < 39.5 Gy and V38Gy < 2 cc. Seymour et al. [21] emphasized the importance of moderate doses and demonstrated the relationship between the V19Gy < 15 cc dose parameter and GU toxicity. Fuji et al. [24] found that acute toxicity was associated with medium-dose regions and late toxicity was associated with high-point-dose regions. However, there are also studies that fail to show the relationship between bladder doses and urinary quality of life (QoL) [27, 28]. This may show us that patient characteristics are also crucial for bladder toxicity [32]. Our study focused solely on the dosimetric components. This should be taken into consideration when evaluating the results.

Regarding GI toxicity, we found that moderate-dose regions were important for both acute and late toxicity. Accordingly, adhering to the recommended doses of V18Gy < 23.02%, V18.12Gy < 22.8%, and V28Gy < 9.6% might potentially decrease acute and late GI toxicity. The HU protocol, PACE‑B, and NRG GU-005 included two dose parameters with similar clinical implications: V18Gy < 35% and V18.12Gy < 50%. Similar to acute GU parameters, we might need a stricter threshold. Only HU protocols included the V28Gy, and our study validated the threshold of below 10% for this parameter. 

GI toxicity is commonly correlated with high-dose parameters and maximum doses [20]. Alayed et al. [23] emphasized the importance of rectum Dmax < 40.6 Gy and V38Gy < 2.1 cc for late GI toxicity. Wang et al. [27] associated the rectum Dmax dose with 1‑month and 2‑year QoL. Elias et al. [28] further highlighted the significance of small volumes and point doses and showed that rectal D1cc > 35 Gy was effective for QoL. Fuji et al. [24] showed, similar to our study, that moderate doses may also be important in acute GI toxicity (D10cc < 25.4 Gy). Qi et al. [22] could not find a relationship between GI dose and QoL. Similarly, our study found no dose parameters that could predict acute grade 1–2 and late grade 3–4 GI toxicity. This might be due to the low number of acute grade 3–4 events. Again, the lack of patient characteristics that could potentially impact acute GI toxicity may have hindered the identification of significant dosimetric parameters.

The second part of our study investigated the effect of prostate volume on toxicity. Currently, the widely recognized threshold of 50 cc for prostate SABR has mostly been extrapolated from brachytherapy experience [13, 14]. However, this threshold was validated in some SABR studies [21, 24]. Still, the absence of high benchmark evidence for this threshold raises concerns about the possibility of overestimated toxicity, perhaps resulting in excessive and protracted use of neoadjuvant ADT, which goes against current standards [33]. Our study suggested that volumes over 60 cc may be significant for acute grade 3–4 GU toxicity. This may be the reason why Janowski et al. [17] safely applied SABR without increased toxicity in patients with a median prostate volume of 62 cc. When planning SABR, it is important to carefully assess individuals with a prostate volume exceeding 50 cc before prescribing neoadjuvant ADT. Treatment should be continued, particularly if the prostate volume is up to 60 cc, as long as it aligns with the goals of the treatment plan.

The most powerful aspect of our study is that it has the largest number of patients among single-center homogeneously treated series using five fractions in the literature. Second, we implemented a highly rigorous monitoring program for the patients, resulting in minimal instances of missing data pertaining to toxicity. Again, the toxicity assessment was carried out under the supervision of the professors in charge. Thus, we tried to minimize the biases introduced by the retrospective nature of the study. Lastly, our study examined other protocols, and all values that were predictive for both GI and GU acute and late toxicities were available in the HU protocol. For this reason, our study not only evaluated the competence of current dosimetric protocols but also added a new protocol to the literature that is predictive for all toxicities.

Our study is not without limitations. A fundamental drawback of our toxicity evaluation is that it relied solely on physician-reported toxicity outcomes. Also, our median follow-up period was limited. Unfortunately, our study was unable to evaluate urethral doses or sexual function. As stated before, our study evaluated only dosimetric parameters; however, dosimetric predictors are not the only determinants of GI or GU toxicity. Last but not least, given the assessment of numerous DVH parameters across various protocols, our results should be evaluated taking into account the multiple-comparisons problem.

## Conclusion

Our study identified that moderate-dose regions play a critical role in both acute and late GI and GU toxicity. For GU toxicity in particular, moderate-dose regions were predictive for grade 1–2 toxicity, while high-dose regions were predictive for grade 3–4 toxicity. The HU protocol encompassed all dosimetric factors found to significantly influence toxicity outcomes, but treatment plans should be evaluated more rigorously as fraction doses increase. Consequently, we asserted that the HU protocol alone is adequate for prostate SABR plans. Additionally, there was a notable correlation between prostate volume and acute grade 3–4 toxicity, suggesting that caution should be taken for prostate volumes exceeding 60 cc. While our study has conducted internal validation of its own protocol, external validation through larger and prospective series is warranted.

## Supplementary Information

Additional data supporting the study’s findings. Supplementary Table 1 outlines the different treatment protocols and organs at risk dose–volume constraints used in SABR plans. Supplementary Table 2 presents descriptive statistics for dose–volume histogram parameters. Supplementary Fig. 1 illustrates toxicity evolution during the follow-ups. Supplementary Fig. 2 shows receiver operating characteristic (ROC) curves for dosimetric variables significant for toxicity in multivariate logistic regression analyses, including various bladder and rectum dose parameters for both acute and late toxicity outcomes.

## Data Availability

The datasets generated and analyzed during the current study are not publicly available but are available from the corresponding author (G. Ozyigit) upon reasonable request.
