# Supplementary material for: Predicting acute and late toxicity in prostate cancer stereotactic ablative radiotherapy: the role of dosimetric parameters and prostate volume
Source: Strahlenther Onkol. 2025 Jan 10;201(8):788–98. doi: 10.1007/s00066-024-02343-2 (PMC12283893; doi:10.1007/s00066-024-02343-2)
Supplement: Supplementary file 1 — Additional data supporting the study’s findings. Supplementary Table 1 outlines the different treatment protocols and organs at risk dose–volume constraints used in SABR plans. Supplementary Table 2 presents descriptive statistics for dose–volume histogram parameters. Supplementary Fig. 1 illustrates toxicity evolution during the follow-ups. Supplementary Fig. 2 shows receiver operating characteristic (ROC) curves for dosimetric variables significant for toxicity in multivariate logistic regression analyses, including various bladder and rectum dose parameters for both acute and late toxicity outcomes. [file 66_2024_2343_MOESM1_ESM.docx]

**Supplementary Material**

**Supplementary Table 1. Different treatment protocols and organs at risk dose-volume constraints used in SABR plans**

|  | **XX-1** | **XX-2** | **PACE-B** | **NRG GU-005** | **RTOG 0938** |
| --- | --- | --- | --- | --- | --- |
| Rectum | D 0.1 cc < 38,06 Gy | D 1% < 35 Gy | V 36 Gy < 1 cc | V 34.4 Gy < 3 cc | D 1 cc ≤ 38.06 Gy |
| Rectum | D 1 cc < 36.25 Gy | D 15% < 32.5 Gy | V 29 Gy < 20% | V 32.63 Gy < 10% | D 3 cc ≤ 34.4 Gy |
| Rectum | D 5 cc < 34.43 Gy | D 40% < 17.5 Gy | V 18.1 Gy < 50% | V 29 Gy < 20% | D 10% ≤ 32.63 Gy |
| Rectum | D 10 cc < 32.62 Gy |  |  | V 18.12 Gy < 50% | D 20% ≤ 29 Gy |
| Rectum | D 20 cc < 25 Gy |  |  |  | D 50% ≤ 18.13 Gy |
| Rectum | V 32 Gy < 5% |  |  |  |  |
| Rectum | V 28 Gy < 10% |  |  |  |  |
| Rectum | V 18 Gy < 35% |  |  |  |  |
| Rectum | V 17.5 Gy < 41 % |  |  |  |  |
| Bladder | D 0.1 cc < 38.06 Gy | D 1% < 35 Gy | V 37 Gy < 5 cc | V 18.12 < 10% | D 1 cc ≤ 38.06 Gy |
| Bladder | D 1 cc < 36.25 Gy | D 5% < 32.5 Gy | V 18.1 Gy < 40 % | V 38.06 Gy < 0.03 cc | D 10% ≤ 32.63 Gy |
| Bladder | D 15 cc < 32.62 Gy | D 10% < 30 Gy |  |  | D 50% ≤ 18.13 Gy |
| Bladder | V 18.1 Gy < 40% | D 35% < 20 Gy |  |  |  |
| Bladder | V 17.5 Gy < 43 % |  |  |  |  |

**Abbreviations:** XX= XXXX University protocol, cc=cubic centimeter, Dx%=dose received by x% of the volume, Dxcc= dose received by x cc of the volume, VxGy= volume receiving a dose X≥ Gy.

**Supplementary Table 2.** Descriptive statistics of dose-volume histogram parameters

| **Parameter** | **Median (range, min-max)** |
| --- | --- |
| **Rectum** | |
| D1% | 34.36 Gy (29.94-37 Gy) |
| D10% | 25. 70 Gy (16.61-30.71 Gy) |
| D15% | 21.59 Gy (12.63- 28.79 Gy) |
| D20% | 18.46 Gy (8.81-25.88 Gy) |
| D40% | 11.31 Gy (4.28-20.76 Gy) |
| D50% | 8.87 Gy (0-18.66 Gy) |
| D 0.1cc | 35.64 Gy (33.37-37.71 Gy) |
| D1cc | 33.61 Gy (26.56-35.50 Gy) |
| D3cc | 30.26 Gy (19.99-33.54 Gy) |
| D5cc | 27.34 Gy (15.93-31.09 Gy) |
| D10cc | 20.74 Gy (6.94-27.40 Gy) |
| D20cc | 13.37 Gy (2.06-21.43 Gy) |
| V17.5Gy | 22.34% (9.15-53.83%) |
| V18Gy | 21.18% (8.44-51.92%) |
| V18.12Gy | 20. 99% (8.28-51.61%) |
| V28Gy | 7.5% (2.17-26.76%) |
| V29Gy | 6.29% (1.70-14.42%) |
| V32Gy | 3.57% (0.57-8.39%) |
| V32.63Gy | 2.68% (0.35-8.12%) |
| V33Gy | 1.41% (0.16-3.50%) |
| V33.5Gy | 1.12% (0.07-3.02%) |
| V34.4Gy | 0.60% (0-2.14%) |
| V36Gy | 0.02% (0-0.68%) |
| **Bladder** | |
| D1% | 35.32 Gy (31.73-37.71 Gy) |
| D5% | 27.55 Gy (11.85-34.63 Gy) |
| D10% | 18.89 Gy (3.19-29.06 Gy) |
| D35% | 3.56 Gy (0.79-14.95 Gy) |
| D50% | 1.55 Gy (0.54-10.21 Gy) |
| D0.1cc | 36.77 Gy (35.47-38.32 Gy) |
| D1cc | 36.14 Gy (34.11-38.07 Gy) |
| D15cc | 25.57 Gy (9.05-33.52 Gy) |
| V17.5Gy | 11.20% (3.62-29%) |
| V18.1Gy | 10.66% (3.49-27.85%) |
| V18.12Gy | 10.62% (3.49-27.71%) |
| V35Gy | 3.69% (0.03-9.75%) |
| V37Gy | 0.01% (0-3.58%) |
| V38.06Gy | 0% (0-1.04%) |

**Abbreviations:** Dx%=dose received by x% of the volume, Dxcc= dose received by x cc of the volume, VxGy= volume receiving a dose X≥ Gy.

**Supplementary Fig. 1** **Toxicity change during the follow-ups**

**Abbreviations:** GU= genitourinary, GI= gastrointestinal

**Supplementary Fig. 2 Receiver operating characteristic (ROC) curves of the dosimetric variables that are significant for toxicity in multivariate logistic regression analyses, a: bladder D10% for acute grade 1-2 GU toxicity, b and c: bladder D1% and D15 cc for acute grade 3-4 GU toxicity, d and e: rectum V18 Gy and V18.12 Gy for GI grade 3-4 toxicity, f: bladder D1% for late grade 1-2 GU toxicity, g: bladder D15cc for late grade 3-4 toxicity, h: V28 Gy for late grade 1-2 GI toxicity**
